# Neutrophil depletion enhanced the *Clostridium novyi*-NT therapy in mouse and rabbit tumor models

**DOI:** 10.1093/noajnl/vdab184

**Published:** 2021-12-21

**Authors:** Verena Staedtke, Tyler Gray-Bethke, Guanshu Liu, Eleni Liapi, Gregory J Riggins, Ren-Yuan Bai

**Affiliations:** 1 Department of Neurology, Johns Hopkins University School of Medicine, Baltimore, Maryland, USA; 2 F.M. Kirby Research Center for Functional Brain Imaging, Kennedy Krieger Institute, Baltimore, Maryland, USA; 3 Department of Radiology, Johns Hopkins University School of Medicine, Baltimore, Maryland, USA; 4 Department of Radiology and Radiological Science, Johns Hopkins University School of Medicine, Baltimore, Maryland, USA; 5 Department of Neurosurgery, Johns Hopkins University School of Medicine, Baltimore, Maryland, USA

**Keywords:** cancer, *Clostridium novyi*-NT, hypoxia, neutrophil, oncolytic therapy

## Abstract

**Background:**

Hypoxia is a prominent feature of solid tumors and can function as fertile environment for oncolytic anaerobic bacteria such as *Clostridium novyi*-NT (*C. novyi*-NT) where it can induce tumor destruction in mice and patients. However, two major obstacles have limited its use, namely the host inflammatory response and the incomplete clearance of normoxic tumor areas.

**Methods:**

In this study, we first used a subcutaneous tumor model of a glioblastoma (GBM) cell line in immunocompetent mice to investigate the local distribution of tumor hypoxia, kinetics of *C. novyi*-NT germination and spread, and the local host immune response. We subsequently applied the acquired knowledge to develop a *C. novyi*-NT therapy in an orthotopic rabbit brain tumor model.

**Results:**

We found that local accumulation of granular leukocytes, mainly neutrophils, could impede the spread of bacteria through the tumor and prevent complete oncolysis. Depletion of neutrophils via anti-Ly6G antibody or bone marrow suppression using hydroxyurea significantly improved tumor clearance. We then applied this approach to rabbits implanted with an aggressive intracranial brain tumor and achieved long-term survival in majority of the animals without apparent toxicity.

**Conclusion:**

These results indicated that depleting neutrophils can greatly enhance the safety and efficacy of *C. novyi*-NT cancer therapy for brain tumors.

**Key Points**

- Neutrophil depletion enhanced efficacy of *C. novyi*-NT oncolytic therapy.
- *C. novyi*-NT therapy was able to cure brain tumors in an orthotopic rabbit model.

**Importance of the Study**

Bacterial oncolytic therapies, especially the ones targeting tumor hypoxia such as *C. novyi*-NT, often encounter incomplete tumor clearance in less hypoxic tumoral areas and severe inflammatory reactions. In this study, we explored immune-modulating preconditioning to suppress the host neutrophils and significantly enhanced the antitumor efficacy of *C. novyi*-NT in animal models, including an orthotopic brain tumor model in rabbits. The optimized preconditioning agent, hyroxyurea, is clinically approved and *C. novyi*-NT has demonstrated manageable safety and promising antitumor responses in clinical trials. Thus, the proposed preconditioning of neutrophil suppression is readily translatable to patients undergoing *C. novyi*-NT trials or other oncolytic biologic therapies and could improve outcome.

Hypoxia in solid tumors is a major hindrance in cancer therapy, as most tumors, including the most aggressive brain tumor, glioblastoma, contain large, poorly vascularized hypoxic areas that limit the efficacy of radiation and chemotherapeutic drugs.^1,2^ However, the presence of hypoxia in solid tumors offers the potential for therapeutic anaerobic bacterial colonization in which anaerobic bacteria destroy the oxygen-low tumor tissue while sparing the well-oxygenated healthy tissues.

A number of bacteria species in genera such as *Salmonella*, *Klebsiella*, *Escherichia*, *Caulobacter*, *Listeria*, *Bifidobacterium*, *Clostridium*, *Streptococcus*, *Lactobacillus*, *Mycobacterium,* and *Proteus* have been developed as oncolytic bacteria.^3^ One successful strain is the anaerobic *Clostridium novyi*. The genus *Clostridium* was shown to cause tumor regression in rodent models, but a subsequent clinical trial failed to demonstrate any clinical benefit in humans that would outweigh the toxic effects.^4^ This concept has been reevaluated using the attenuated *C. novyi*-NT strain, which is characterized by a deletion of the lethal toxin gene rendering the bacterium less toxic. We and others have shown that *C. novyi*-NT can selectively germinate and grow in the hypoxic regions of solid tumors after intravenous injection with less toxicity.^4–7^ More recently, we also showed that intratumoral injection of *C. novyi*-NT spores may be a promising approach to facilitate germination and reduce spore dosage needed for germination.^8^ However, the challenges of *C. novyi*-NT cancer therapy remain to be substantial with severe host inflammatory responses and reduced efficacy in clearing up tumor outer rims.^4^

In this study, based on the observation of leukocyte accumulation around the germination in rat brain tumor, we utilized different methods of neutrophil depletion and evaluated the impact on toxicities and tumor clearance.^7^ We focused on the deadly glioblastoma, a malignancy arising from the glial cells in the brain, for which conventional treatment options such as chemotherapy and radiation therapy are limited, with an average life span of 1-2 years.^9^

When such therapy is applied to the brain, edema might occur due to inflammatory infiltrates, which within the fixed size of the cranial vault may cause fatally raised intracranial pressure. This is particularly challenging in rodents, where the cranial volumes are small (mouse ~440 mm^3^ and rat ~1200 mm^3^) and animals can easily succumb to brain swelling during germination. For example, the *C. novyi*-NT germination in intracranial GL261 mouse brain tumor can lead to death of the majority of the mice in our experience (unpublished observation). We thus first developed the neutrophil depletion approach using an immunocompetent subcutaneous mouse glioblastoma tumor model and subsequently tested it in a rabbit brain tumor model, whose brain size around 12,000 mm^3^ offers significantly more space to accommodate the swelling than that of a mouse.

## Material and Methods

### Cell Lines and Tissue Culture

The mouse GL261 glioma cell line was obtained from the German Collection of Microorganisms and Cell Cultures (DSMZ), Germany, and was authenticated by DSMZ. It was maintained in Dulbecco’s Modified Eagle Medium (DMEM) media supplemented with 10% fetal bovine serum (FBS) and antibiotics and GL261-luc cells were generated by infection of lentivirus carrying luciferase as described.^10^

## Tumor implantation

### Subcutaneous GL261 Tumor

All methods of animal experiments used in this study were in accordance with standards set by the JHU animal care and use committee (ACUC).

As we observed an unstable intake rate of subcutaneous GL261 tumor implanted directly from cultured cells into the syngeneic C57BL6 mice, we used serial passaging to maintain the GL261 tumor. To generate the initial tumor, 5 × 10^6^ GL261-luc cells in 0.1ml were mixed with equal volume of Matrigel Matrix (BD, Cat. No. 354248) on ice and injected subcutaneously in the flank of a female 6-8 weeks old C57/BL6 mouse. Subsequently, the tumor was dissected into 2-3 mm pieces and serially transplanted subcutaneously to generate further flank tumors.

### Intracranial VX2 Tumor

Male rabbits of 4–6 lbs. were purchased from Robinson Services Inc. Rabbit VX2 tumor was established from a carcinoma induced by the Shope cottontail rabbit papillomavirus (CRPV)^11^ and passaged via hind limb donor rabbit as described previously.^12^ Briefly, a vial of frozen VX2 cells was defrosted in warm water, centrifuged, and injected in the hind leg muscle of a donor New Zealand White rabbit to allow several weeks for the tumor to grow. Rabbits were anesthetized by intramuscular injection of ketamine (50 mg/kg) and xylazine (5–10 mg/kg) and a viable part of tumor within the proliferative rim was excised, minced to small pieces using a scalpel, and dissociated with 1:2 mixture of collagenase (10 mg/ml) and hyaluronidase (1000 unite/ml) in PBS in a flask at 37°C and 200 rpm rotation. Tumor cells were counted and frozen in DMEM/F12 media supplemented with 10% FBS, antibiotics, and 10% DMSO, or used for implantation subsequently. For intracranial implantation, 5 × 10^5^ VX2 cells in 1.5 µl DMEM/F12 media were injected 10 mm deep, 4 mm right and 3 mm anterior of the bregma. To prevent tumor cell seeding from the needle tract on the brain surface and the meninges around the burr hole, the burr hole was rinsed with 0.5 µg/ul doxorubicin in PBS after needle retraction and inserted with a 3 mm gel stick prepared with 0.25 µg/ul doxorubicin in PBS as described in our previous study.^13^ The burr hole was sealed by bone wax and the scalp was sutured together. Rabbits were monitored for stress signs in eating and fecal patterns in accordance with the JHU ACUC protocol.

## Preparation and Intratumoral (IT) Injection of *C. novyi*-NT Spores

### Spore Production and Purification

C. *novyi*-NT spores were produced and purified as previously described.^8,14^ Briefly, bacteria were grown in sporulation medium for two weeks and mature spores were purified through two consecutive Percoll gradients followed by four washes and re-suspensions in PBS. Spores were tested for sterility by culturing product in Soybean-Casein Digest Medium and Thioglycollate Medium (Nelson Laboratories, Salt Lake City, UT). Germination efficiency assays were performed on Brucella agar with 5% horse blood. Spores were stored at a concentration of 3 × 10^9^ spores/ml in sterile PBS at 4° C.

### Flank GL261 Tumor Treatment

Mice were randomized after tumor implantation before assigned to the control and treatment groups. Prior to IT injection, spores were thoroughly re-suspended with a vortex and taken up in a 10 µl Hamilton syringe with a 32G needle. For the flank GL261 tumor, the injection site was aseptically prepared and a total of 3 µl spores at 6 × 10^6^/µl were injected in three perceived center locations. The spores were concentrated to reduce the injection volume to minimize the disturbance of local tumor hypoxia and multiple locations were intended to ensure the placement of spores in tumor hypoxic areas. The injection needle was held for 30 sec and removed slowly and the injection site sterilized. To label the tumor hypoxia, GL261 tumor-bearing mice were injected with pimonidazole hydrochloride (PMN, Hydroxyprobe) IP at 60 mg/kg 60 min prior to tumor harvesting and the tumors were preserved immediately in 10% formalin at RT.

### Intracranial VX2 Tumor Treatment

Rabbits were randomized after tumor implantation before assigned to control and treatment groups. Six days prior to the spore injection, in the HU group, hydroxyurea (HU, Abcam) dissolved in PBS was injected at a dose of 500 mg/kg subcutaneously daily. On the day of spore injection, HU dose was reduced to 100 mg/kg and repeated for another 7 days.

Three days prior to the spore injection, in the CPA group, cyclophosphamide (TCI Chemicals) dissolved in PBS was injected IP at the dose of 125 mg/kg as one-dose pretreatment along with 50 mg/kg Mesna to mitigate CPA’s potential urotoxicity.

On day 17 after VX2 tumor implantation, VX2-bearing rabbits were anesthetized and injected with 1 µl *C. novyi*-NT spores at 6 × 10^6^/µl in 8.5 mm depth and another 0.5 µl in 6.5 mm depth during needle retraction through the existing burr hole. Dexamethasone sodium phosphate (Matrix Scientific) was injected at a dose of 5 mg/kg subcutaneously at the time of spore injection and repeated daily in the following two days to minimize the risk of edema. Rabbits were observed closely for any signs of deterioration, lethargy, neurotoxicity, or pain in accordance with the Johns Hopkins Animal Care and Use Guidelines.

### Hematological Analysis

Mouse and rabbit blood were mixed with 5 mM EDTA and analyzed by a ProCyte Dx Hematology Analyzer.

### Immunohistochemistry and Immunofluorescence Staining

Tumors and rabbit brains were preserved in 10% formalin and paraffin sections were obtained. Gram-stained slides, counter-stained with safranin, and H&E-slides were prepared according to routine histopathologic practices. Paraffin section of tumors were deparaffinized, rehydrated, and antigen-retrieved using the Citra buffer (Biogenex) as described.^15^ Immunohistochemistry (IHC) staining of PMN-labeled sections followed the procedure established before using monoclonal Hypoxyprobe-1 antibody (Hypoxyprobe Inc.), biotin-conjugated F(ab’)2 (JacksonImmunoResearch, 315-066-047) and streptavidin peroxidase (Biogenex HK330-9KT).^16^

Immunofluorescence staining was performed using mouse anti-Ly6G 1A8 antibody (BD), mouse anti-PMN Hypoxyprobe-1 antibody (Hypoxyprobe Inc.), or rabbit anti-*C. novyi*-NT antiserum that was obtained from a rabbit with *C. novyi*-NT germination in the leg VX2 tumor. Antimyeloperoxidase (MPO) antibody (R&D Systems, AF3667) was used in IHC with biotin-conjugated donkey antigoat F(ab’)2 (JacksonImmunoResearch, 705-066-147) and streptavidin peroxidase (Biogenex HK330-9KT). Antirabbit Alexa 488 and antimouse Alexa 594 (Invitrogen) secondary antibodies were applied and 4′,6-diamidino-2-phenylindole (DAPI, Vector Laboratories) stained the nuclei, following the procedure described previously.^15^

### Statistical Analysis

Results are presented as a mean value ± standard deviation. P values were determined by a Mantel-Cox test and *P* values < 0.05 were deemed as statistically significant. Survival was plotted on Kaplan-Meier curve. Data were analyzed by GraphPad Prism, version 5.0.

## Results

### Neutrophil Accumulation and Depletion in *C. novyi*-NT Therapy of Subcutaneous GL261 Tumor

**Figure 1.**
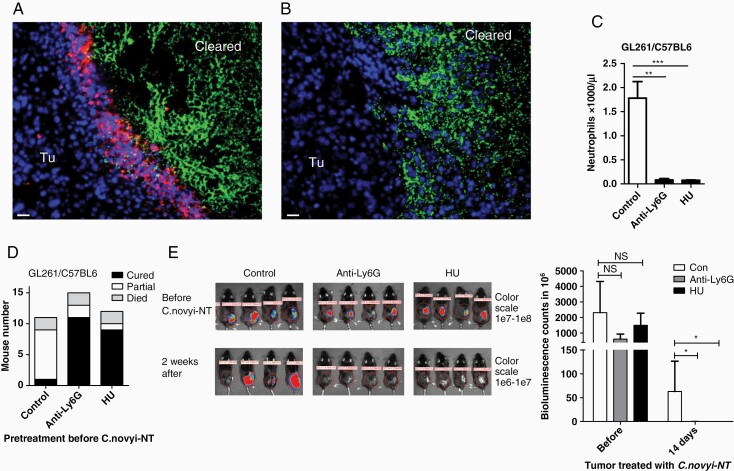
Neutrophil barriers prevented tumor clearance by *C. novyi*-NT in a subcutaneous mouse tumor model. A. Flank GL261 tumor was injected intratumorally with *C. novyi*-NT spores and harvested 12 h later, fixated and stained with anti-Ly6G (1A8, red) and anti-*C. novyi* (green) antibodies. Neutrophils were shown of forming a barrier between the tumor and germinating *C. novyi*-NT. Cleared: area where tumor cells were completely eradicated. Scale bar: 20 µm. B. Mouse bearing flank GL261 tumor was treated with anti-Ly6G antibody intraperitoneally (IP) 24 hours prior to the injection of *C. novyi*-NT spores. Tumor was harvested and stained as described in A. Neutrophil accumulation was not observed with the germinating bacteria. Scale bar: 20 µm. C. Blood neutrophil count in GL261 tumor-bearing mice treated with anti-Ly6G antibody (48 h) or HU (5 days) were measured and compared with the untreated control mice, showing the treatments greatly suppressed the neutrophil levels. *n =* 3. D. Efficacies of intratumoral *C. novyi*-NT treatment in flank GL261 tumor. Suppressing neutrophils via anti-Ly6G or HU significantly reduced the partial tumor clearance and improved the total tumor clearance (cured). The occurrence of treatment-related death remained similar among the groups (died). E. An example of bioluminescence monitoring of luciferase-labeled flank GL261 tumors in different treatment groups as described in D (left panels). Bioluminescence counts were quantified and analyzed for statistical significance using T test (right graph). *n =* 4.

The neutrophils are the effector cells of the innate immunity and a major phagocyte in host defense against bacterial pathogen. Neutrophils migrate to the site of infection and use a combination of antimicrobial utilities such as cytotoxic granule contents, antimicrobial peptides, reactive oxygen species (ROS), and neutrophil extracellular traps (NETs) to generate a highly lethal antibacterial environment.^17^ In our study, mouse GL261 glioma cells were implanted in C57BL6 mice subcutaneously and when the tumors reached the sizes of 400–500 mm^3^, 2 × 10^7^*C. novyi*-NT spores were injected into three different central areas of the tumor body. This tumor size was selected to ensure consistent *C. novyi*-NT germination as we observed inconsistent germination in smaller tumor in this model (data not shown). Mice were observed for tumor swelling and hemorrhagic necrosis, and signs for toxicity. Tumors were harvested 24 hours after the spore injection and staining via H&E and Gram demonstrated extensive germination, accompanied by marked accumulation of polymorphonuclear granulocytes located between the germinating bacteria and remaining viable tumor, mainly around the outer rim area (Supplementary Figure 1A). The cell membrane antigen Ly6G is expressed in granulocytes and transiently in some monocytes and is a well-defined marker for murine neutrophils, especially the circulating and recruited neutrophils.^18^ Immunofluorescence staining of 1A8 anti-Ly6G antibody (red) identified the majority of those cells as neutrophils (Figure 1A). Those immune cells could be depleted by treating the mice with 1A8 antibody intraperitoneally (IP) 24 hours before the spore injection (Figure 1B, Supplementary Figure 1B). Staining with anti-CD11b and CD3 antibodies (red) revealed minimal numbers of positive cells among those accumulated immune cells (Supplementary Figure 2A & B). Similar depletion was achieved also by daily IP injection of hydroxyurea (HU), an immunosuppressant used in treating myeloproliferative diseases, for 5 days before the spore injection, as indicated by the complete blood count (CBC) of mouse blood (Figure 1C). The counts of blood cells with the suppression by HU showed also a significant drop of lymphocytes (Supplementary Figure 2C). Previous studies have demonstrated substantial mortality rates of *C. novyi*-NT-treated mice with large subcutaneous CT26 and GL261 tumors (~700 mm^3^ or 600-900 mm^3^).^5,19^ In this study, we used moderate tumor sizes and while germination occurred in all tumors, 2 out of 11 mice (2/11) died of treatment-related toxicity within 48 hours. Of the surviving animals, 8 mice with initial tumor reduction were found later to have tumor regrowth (partial), and 1 mouse was cured as determined by absence of tumor after 3 weeks (Figure 1D). In contrast, in the cohorts treated with anti-Ly6G 1A8 antibody or HU, 11/15 or 9/12 mice respectively were cured of tumor, 2/15 or 1/12 were partial, and 2/15 or 2/12 died from immediate toxicities, respectively (Figure 1D). HU or 1A8 antibody alone did not show any noticeable therapeutic effects (data not shown). Bioluminescence imaging of the luciferase-labeled tumor illustrated these therapeutic outcomes (Figure 1E).

### Tumor Hypoxia and a Model of *C. novyi*-NT Germination

**Figure 2.**
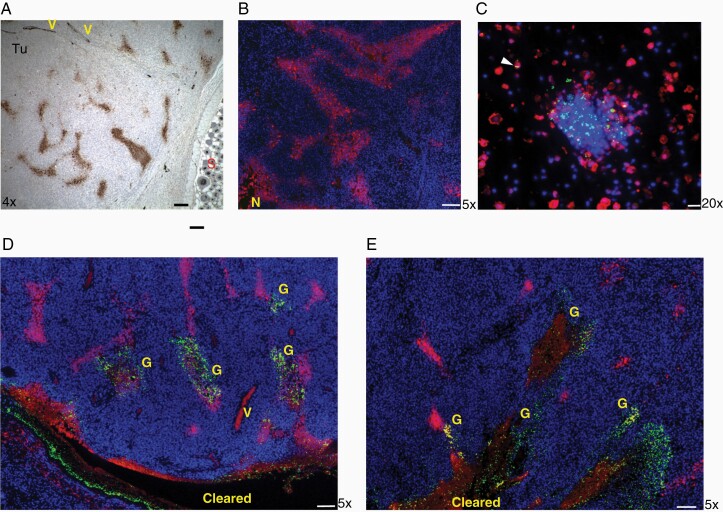
Distribution of tumor hypoxic areas and *C. novyi*-NT germination. A&B. Mice bearing flank GL261 tumor were injected with pimonidazole (PMD) 60 min before harvesting. Hypoxia was visualized by immunohistochemistry (IHC, brown) in A or immunofluorescence (IF, red) in B via the mouse Hypoxiprobe antibody. Hypoxic pockets were observed in the tumor outer rim area. S: surrounding tissue. Scale bar: 100µm. C. A presumed injection site of *C. novyi*-NT spores was analyzed by anti-PMD Hypoxyprobe-1 antibody (red), anti-*C. novyi* antibody (green) and DAPI (blue). A mix of spore (small round) and germinated (rod) forms of *C. novyi*-NT was observed. The white arrow head indicated a germinated *C. novyi*-NT within a hypoxic tumor cells. Scale bar: 20 µm. D&E Macroscopic views of *C. novyi*-NT germination in flank GL261 tumor labeled with PMD and pretreated with anti-Ly6G 24 hours before. IF staining showed germination (G, green) largely occurred within the tumor hypoxic areas (red). Red blood cells in the vessels also appeared red. Cleared: area where tumor cells were completely eradicated. V: blood vessels. Scale bar: 100 µm.

**Figure 3.**
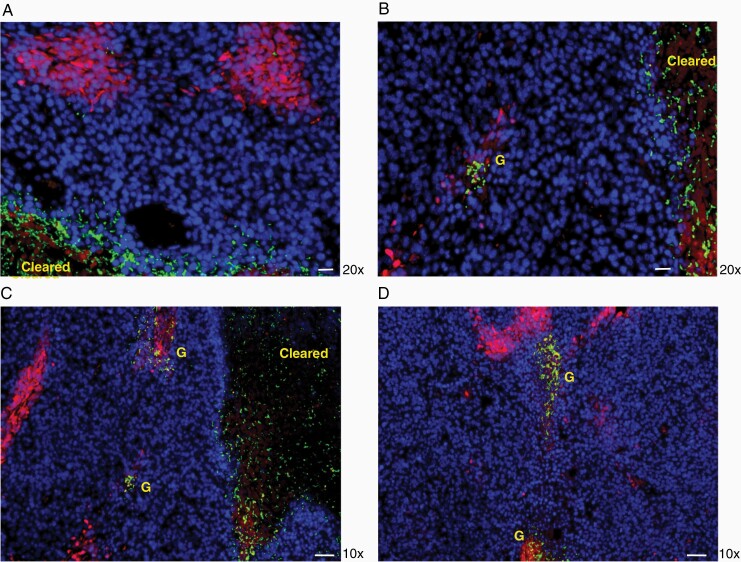
Spreading of the *C. novyi*-NT germination in the tumor followed the tumor hypoxic areas. A. Spreading of individual *C. novyi*-NT bacteria (green) in the tumor body adjacent to a main germination pocket free of tumor cells (cleared). Scale bar: 20 µm. B. Germination (G, green) occurred within a hypoxic area adjacent to a main germination pocket cleared of tumor cells (cleared). Scale bar: 20 µm. C&D. Germination (G, green) within the hypoxic areas within a tumor. A germinating area free of tumor cells (cleared) was shown in C, with adjacent smaller hypoxic pockets containing germinating bacteria (G, green). Scale bar: 50 µm.

While these results demonstrated the essential role of neutrophil defense in preventing complete tumor clearance by *C. novyi*-NT, they raised the question of how the clearance of whole tumor was achieved as the tumor outer rim areas, which are distant from the necrotic centers and close to blood vessels, are regarded as nonhypoxic in a number of tumor models.^20^ To investigate the spatial distribution of hypoxia in the flank GL261 tumor, we labeled the tumor with the hypoxia indicator pimonidazole (PMN) via IP injection 90 min before harvesting the tumor. Immunohistochemistry staining of PMN using Hypoxyprobe-1 monoclonal antibody (Mab, brown), which stains tissues with an oxygen pressure below 10 mmHg,^21^ showed a distribution of small hypoxic pockets in the tumor body and rim areas adjacent to surrounding tissues (Figure 2A). Some apparent tumor vessels were also stained positive of bound PMN as exemplified in Figure 2A and Supplementary Figure 3 (brown). This distribution pattern of tumor hypoxia was also illustrated by the immunofluorescence (IF) staining of PMN using the same Hypoxyprobe-1 Mab (red) (Figure 2B). Next, we attempted to visualize the germination process in a time course after *C. novyi*-NT spore injection in GL261 tumor pretreated with anti-Ly6G antibody for neutrophil depletion. At 6, 8, and 12 hours following spore injection, GL261 tumors were harvested after 1 hr of PMN labeling. No significant germination was observed in the 3 harvested tumors at 6 hours (data not shown). After 8 hours, germination could be determined in some areas of the tumor via IF using rabbit anti-*C. novyi*-NT antibody. In Figure 2C, a presumed initial germination site was observed in higher magnification, where the spores, stained as green dots, were found among cell aggregates which were likely accumulated immune cells, surrounded by hypoxic tumor cells stained in red (Figure 2C). Some germinating bacteria in the rod form were found co-localizing with hypoxic tumor cells (Figure 2C, white arrow head). Macroscopic view of tumors 12 hours after the spore injection revealed a germination pattern, which appeared to spread from one large germinated area (green) to adjacent small hypoxic pockets (red) (Figure 2D&E). Pictures of higher magnifications showed that individual *C. novyi*-NT bacteria (green) were distributed sparsely in the surrounding tumor tissue of a germination area, some of which were found within a hypoxic pocket (red) (Figure 3A) and initial colonization occurred in such hypoxic pockets (Figure 3B-D). Based on this observation, a germination and spreading mechanism has emerged, in which the injected spores germinate first in a hypoxic/necrotic area, then the bacteria powered by flagellum movement migrate randomly into the vicinity to encounter another hypoxic pocket where they colonize and form a new germination center. In the absence of the intervention of neutrophils, within several hours, ever-expanding germination fronts supported by hypoxic pockets presented throughout the tumor body and rim led to destruction of the whole tumor.

### *C. novyi*-NT Therapy of Orthotopic VX2 Rabbit Brain Tumor

**Figure 4.**
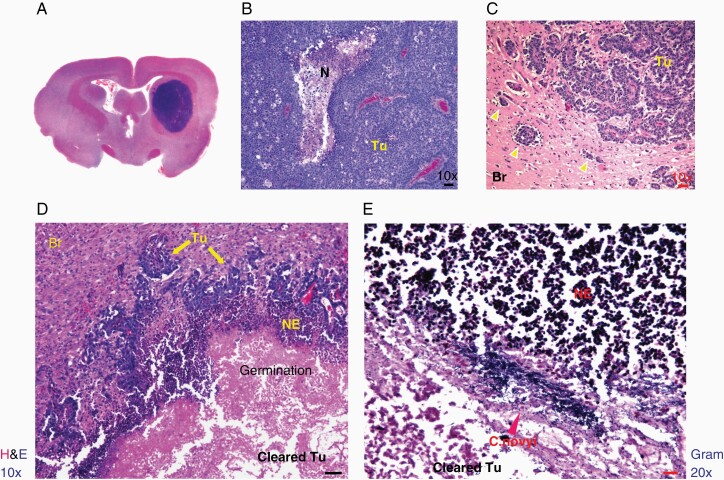
Neutrophil barriers restricted the spread of *C. novyi*-NT in the VX2 rabbit brain tumor. A. H&E staining of a coronal section of a rabbit brain at day 19 after VX2 tumor implantation. Tumor was stained blue in the right frontal lobe. B. H&E staining of the main body of VX2 brain tumor showed dense tumor cells with a necrotic area (N). Scale bar: 50 µm. C. Invading tumor cells (yellow arrow heads) in the brain tissue (Br) were observed around the rim of VX2 brain tumor in the H&E-stained section. This resembles the invasive feature of human glioblastomas. Scale bar: 50 µm. D. Areas of *C. novyi*-NT germination were surrounded by neutrophil layers in the tumor main body that has been cleared of tumor cells (cleared Tu). Scale bar: 50 µm. E. A front of germinating *C. novyi*-NT (blue) visualized by Gram staining was blocked by a layer of neutrophils (NE). Scale bar: 20 µm.

**Figure 5.**
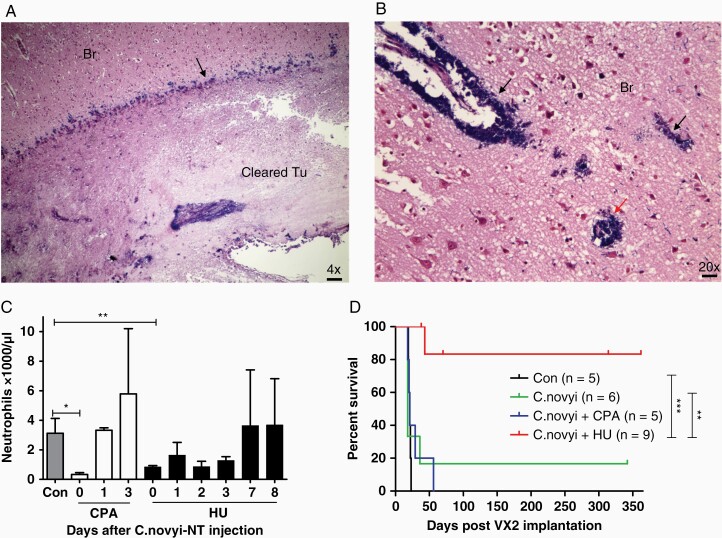
Neutrophil suppression improved the efficacy and safety in rabbit VX2 brain tumor model. A. A macroscopic view of a germinating front (blue, Gram staining) of *C. novyi*-NT and cleared tumor main body in the rabbit VX2 brain tumor 72 hours after spore injection. Scale bar: 100 µm. B. Germinating *C. novyi*-NT in the suspected invading tumor blood vessels (black arrows) and satellites (red arrow) in the rabbit brain (Br). Scale bar: 20 µm. C. Comparing neutrophil suppression by cyclophosphamide (CPA) and hydroxyurea (HU) (*n* = 3–5) by measuring the blood neutrophil counts before and after *C. novyi*-NT spore injection. The baseline neutrophil level without pretreatment was used as control (*n* = 4). Neutrophil levels rebounded 3 days after the *C. novyi*-NT treatment, compared with 1 day in the CPA-pretreated group. D. Neutrophil suppression via HU pretreatment greatly improved tumor clearance and reduced treatment-related toxicity of *C. novyi*-NT in VX2 brain tumor model. Rabbit survival data of control, intratumorally injected *C. novyi*-NT spore alone, *C. novyi*-NT spores with CPA pretreatment, and *C. novyi*-NT spores with HU pretreatment were plotted in Kaplan-Meier survival curves. Median survival: Con = 21 days, *C. novyi*-NT = 18 days, *C. novyi*-NT + CPA = 21 days. Death time: Con (*n* = 5): day 19, 20, 21, 22, 23; *C. novyi*-NT (*n* = 6): day 18, 18, 18, 18, 36, 1 long-term survivor; *C. novyi*-NT + CPA (*n* = 5): day 18, 20, 21, 29, 56; *C. novyi*-NT + HU (*n* = 9): day 43, 8 long-term survivors.

Rabbit VX2 tumor was established from a carcinoma induced by the Shope cottontail rabbit papillomavirus (CRPV)^11^ and has been widely used as a rabbit intracranial tumor model for various imaging and therapeutic studies.^22–24^ As detailed in the Introduction, we chose the rabbit model to study *C. novyi*-NT germination for the larger cranial size. VX2 tumor cells were implanted in the right frontal lobe (Figure 4A) and rabbits became symptomatic with a tilted head, severe reduction of eating and fecal output at day 19–23 after the tumor implantation, which necessitated euthanasia, resulting in a median survival of 21 days for untreated animals (Figure 5D). *C. novyi*-NT treatment was initiated relatively late at day 17 after tumor implantation to ensure that a sizable tumor had formed which is needed for IT spore injection to support a robust germination. Histologically, VX2 brain tumor resembled some key features of human glioblastoma, such as necrotic centers and invasive growth pattern observed as small tumor satellites in the surrounding brain tissues (Figure 4B&C). *C. novyi*-NT germination in the tumor led to sizable accumulation of neutrophils that prevented bacteria from completely disseminating to the remaining tumor and particularly the outer rim (Figure 4D&E). Pretreatment of rabbits with HU, similar as observed with GL261 model before, was effective in suppressing the intratumoral accumulation of neutrophils, which led to enhanced bacterial tumor eradication. Gram staining of a brain section from a HU-pretreated rabbit 72 hours after the spore injection revealed an apparent complete tumor clearance with a germinating front (blue) (Figure 5A). Within the surrounding brain tissue, some tumor vessel structures and invading satellites were colonized by *C. novyi*-NT (black and red arrows) (Figure 5B). Due to the lack of available antibody targeting rabbit neutrophils, we compared cyclophosphamide (CPA) and HU for their efficacies in neutrophil depletion. Although both CPA and HU could achieve significant neutrophil suppression, neutrophil counts quickly rebounded within 2 days after spore injection in the CPA cohort, while the rabbits pretreated by HU maintained lower neutrophil counts for at least 3 days after spore injection (Figure 5C), thereby providing a potential therapeutic window for tumor eradication. At the administered dose, the HU-induced neutrophil suppression was pronounced while lymphocyte counts were not significantly affected by HU; however, platelets showed a marked drop (Supplementary Figure 4). Pretreatment with HU significantly improved the therapeutic efficacy and safety of IT *C. novyi*-NT therapy and resulted in 8 long-term survivors out of 9 rabbits, with one death owing to tumor regrowth 26 days after spore injection as determined by autopsy and brain sections (Figure 5D). In comparison, among 6 rabbits injected with *C. novyi*-NT spores alone at day 17 without pretreatment, 4 died from immediate toxicities at day 18, 1 died from tumor regrowth 19 days after spore injection and 1 rabbit achieved long-term survival until euthanized in a healthy condition 342 days after tumor implantation (Figure 5D). The control group died from tumor between day 19–23. The toxicity-related death can be distinguished from tumor-related death through the acute onset and severe progression of the symptoms that required immediate euthanasia. The cohort pretreated with CPA prior to spore injection was inferior to HU and achieved similar results as observed in the *C. novyi*-NT alone cohort without any long-term survivor.

**Figure 6.**
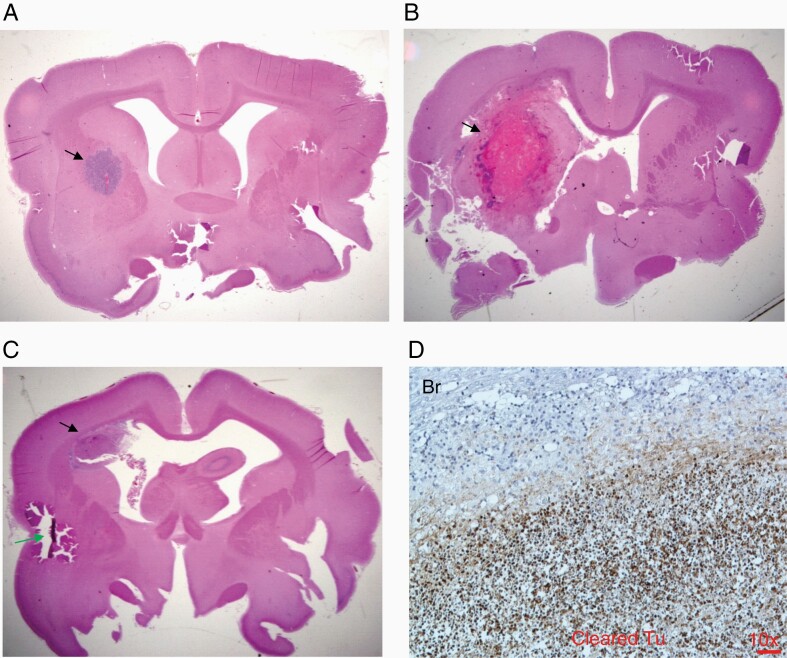
A macroscopic overview of *C. novyi*-NT treated VX2 tumor in the rabbit brain. A. H&E staining of a VX2 tumor at the day of *C. novyi*-NT treatment. Tumor was implanted in the right frontal lobe (black arrow). B. VX2 tumor was stained by H&E after the treatment-related death one day post *C. novyi*-NT spore injection. Severe inflammatory reaction was observed around the tumor site featured by widespread hemorrhage (black arrow). C&D. A rabbit brain was harvested 8 days after the *C. novyi*-NT spores injection in a HU pretreatment rabbit while the animal was in a healthy condition, and stained with H&E or IHC of neutrophil enzyme MPO. The germination area showed a restrained pattern (black arrow) (C) and no tumor cells were observed in the rim of the cleared tumor body. A tear of the section was indicated by the green arrow. Anti-MPO IHC showed that the space was filled with neutrophils (brown) (D). Scale bar: 50 µm.

Next, we closely examined the rabbits’ brain sections in the various treatment regimens. An example of VX2 brain tumor section at the day of *C. novyi*-NT treatment is shown in Figure 6A (black arrow). The brain section of a rabbit that died one day after the spore injection without neutrophil depletion revealed a severely hemorrhagic and expanded lesion (black arrow) with considerable mass effect due to edema compared to the contralateral brain structure (Figure 6B). This stands in contrast to a “transformed” lesion (black arrow) of a HU-pretreated rabbit sacrificed in healthy condition 8 days after spore injection (Figure 6C), which upon closer examination was free of tumor and germinating bacteria but predominantly filled with neutrophils as demonstrated by antimyeloperoxidase (MPO) IHC (brown) (Figure 6D, Supplementary Figure 5A&B). MPO can be released into the surrounding by neutrophils during degranulation to facilitate the killing of bacteria.^25^ Of note, neutrophil counts made a rebound around day 3-7 after the *C. novyi*-NT spore injection (Figure 5C). As a minority of rabbits in the *C. novyi*-NT alone group survived the initial germination and died from tumor regrowth (Figure 5D), we sacrificed another otherwise healthy rabbit on this regimen 8 days after spore injection and observed a restricted lesion filled with neutrophils without germinating bacteria (Supplementary Figure 6A&B), however, pockets of growing tumor in the former tumor-brain transitional areas were discovered, indicating a partial tumor clearance (Supplementary Figure 6C&D).

## Discussion

In adoptive T cell therapies, preconditioning by lymphodepletion has been incorporated in the treatment protocol to achieve optimal efficacies, which includes using chemotherapeutics such as fludarabine and cyclophosphamide or full-body radiation.^26–29^ This is presumably facilitated by eliminating regulatory T cells and competing elements of the immune system, which help prolong the persistence of infused cells. In oncolytic virus therapies, preconditioning with immune modulators or complement depletion successfully improved the viral targeting of tumors.^30,31^ So far, immune-modulating pretreatment has not been used in therapies with oncolytic bacteria.^3^

In this study, we reported the development of a combination therapy of *C. novyi*-NT and neutrophil reduction, with reduced cytotoxicity and substantially enhanced antineoplastic potency in the orthotopic rabbit brain tumor model and with enhanced efficacy in a subcutaneous mouse glioma model. Neutrophils were rapidly recruited to the tumor sites in response to *C. novyi*-NT germination and the accumulation of neutrophils inside the tumor caused a barrier that limited the bacterial spread. Drug-induced suppression or antibody-mediated depletion of neutrophils permitted the bacteria to replicate and spread within the tumors unhindered and subsequently, led to enhanced tumor clearance. Importantly, over 70% of animals treated with such combination showed no evidence of tumor recurrence and remained alive compared to <10% in the control. In the orthotopic rabbit brain tumor mode, HU pretreatment also significantly reduced *C. novyi*-NT-induced toxicity which may be related to the suppression of the myeloid cell populations that are responsible for the release of acute inflammatory cytokines.^19,32^ In animals with tumor recurrence, we suspect that *C. novyi*-NT could not effectively colonize the peritumoral regions, which are less hypoxic. Those well-vascularized tumor regions are susceptible to chemotherapeutic drugs, and thus the combinatorial use of these agents may further improve the treatment efficacy. Although the effectiveness of inflammation suppression to substantially enhance the oncolytic potency was only evaluated in one bacterium, its general applicability to other types of oncolytic bacteria is likely but will need to be validated in relevant animal models. It is worth mentioning that in oncolytic viral therapies, local inflammation and infiltration of immune cells in the tumor tissue are desired and vital for immunogenic killing of the tumor.^33,34^

It is notable that the neutrophil-suppressing pretreatment reduced the toxicity-related death in the orthotopic rabbit brain tumor model (4 out of 6 in *C. novyi*-NT only group vs 0 out of 9 in *C. novyi*-NT plus HU group in Figure 5D), while low but largely unchanged toxicity-related death rates were observed in the subcutaneous GL261 mouse model with or without pretreatment (Figure 1D). The rapid rise of intracranial pressure in the VX2 brain tumor model during germination was likely the main cause of toxicity-related death and a reduction of brain edema indicated by the less inflammatory lesion (Figure 6C) via suppression of neutrophils has a very sensitive effect on mitigating the animal mortality. Less local infiltration and accumulation of neutrophils and possibly other myeloid cells suppressed by HU may have contributed to the edema reduction and improved safety.

Hypoxia in solid tumor arises in regions with insufficient oxygen supply, which, in the general view, is attributed principally to the distance to blood vessels.^20^ However, in tumors such as glioblastoma, where vasculatures are often disorganized and dysfunctional, and a small percentage of vascular endothelium could be of neoplastic origin,^35–37^ local hypoxia may not necessarily be determined by the distance to the blood vessels. Of note, in human glioblastomas and high-grade astocytomas, oxygen pressure was measured at 9.2 ± 5.8 mmHg or 15.3 ± 2.3 mmHg in intratumoral locations, at 17.9 ± 9.3 mmHg in peritumoral areas and at 59.8 ± 6.5 mmHg in the brain tissue,^38,39^ which is consistent with the PMN staining in this study that labels tissues with oxygen pressure below 10 mmHg.^21^*C. novyi* can achieve full germination below the oxygen pressure of 7.6 mmHg and tolerate up to 15.2 mmHg.^40^ The mouse tumor model in this study was established from subcutaneously implanted GL261 glioblastoma cell line and the PMN-mediated staining revealed a wide distribution of relatively small hypoxic pockets throughout the viable tumor, including the tumor rim. The biological functionality of those hypoxic pockets was essentially validated by focused colonization of germinating *C. novyi*-NT bacteria. Interestingly, multiple tumor vascular structures were also stained positive of hypoxia, underlying their dysfunctionality. In fact, the origin of tumor hypoxia may involve multiple and complex factors, including hyper respiratory activity in mitochondria as an intrinsic hypoxia generator in the tumor cells as shown in previous studies.^41,42^ Also demonstrated by those studies was that such intrinsic tumor hypoxia was measured inconsistently and may vary greatly among different types of tumor cells, generally correlating with the aggressiveness of the tumor,^41,42^ consistent with our unpublished observation. This could indeed present a challenge to therapeutics targeting tumor hypoxia, including *C. novyi*-NT, in a broad application of different tumor types. When used in treating aggressive brain tumor, however, *C. novyi*-NT demonstrated promising therapeutic efficacies once we incorporated modulation of immune response to ensure the spread of the bacteria and reduce side effects. These improvements may lead to the development of safer and more effective oncolytic bacterial treatments for patients with glioblastomas and other poorly vascularized tumors in the future.

## Supplementary Material

vdab184_suppl_Supplementary_Figures

vdab184_suppl_Supplementary_Figure_Legends
