# Supplementary material for: Neutrophil depletion enhanced the Clostridium novyi-NT therapy in mouse and rabbit tumor models
Source: Neurooncol Adv. 2021 Dec 21;4(1):vdab184. doi: 10.1093/noajnl/vdab184 (PMC8807082; doi:10.1093/noajnl/vdab184)
Supplement: vdab184_suppl_Supplementary_Figures [file vdab184_suppl_supplementary_figures.pptx]

## Slide 1
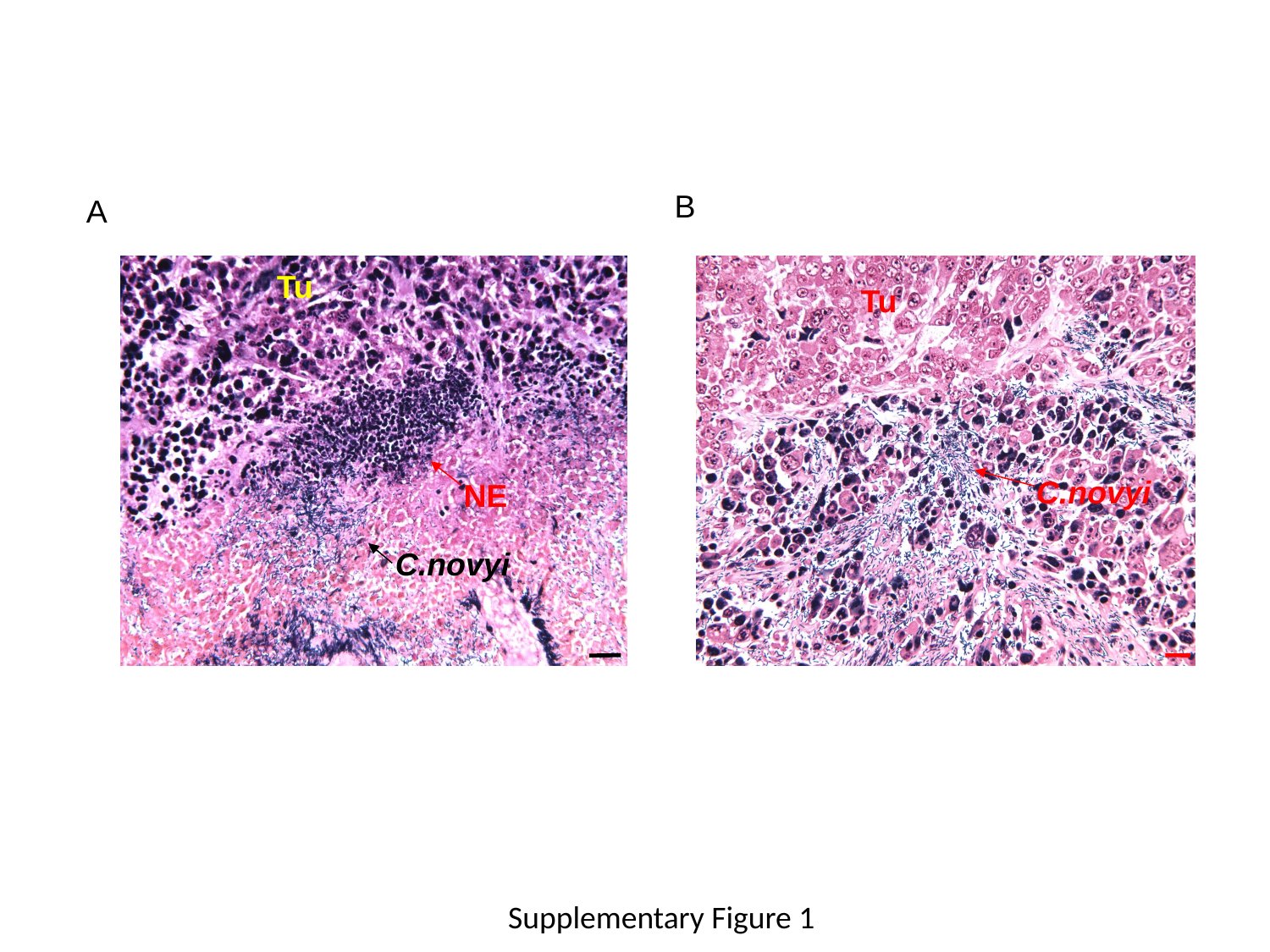

B
A
Tu
Tu
C.novyi
NE
C.novyi
Supplementary Figure 1

## Slide 2
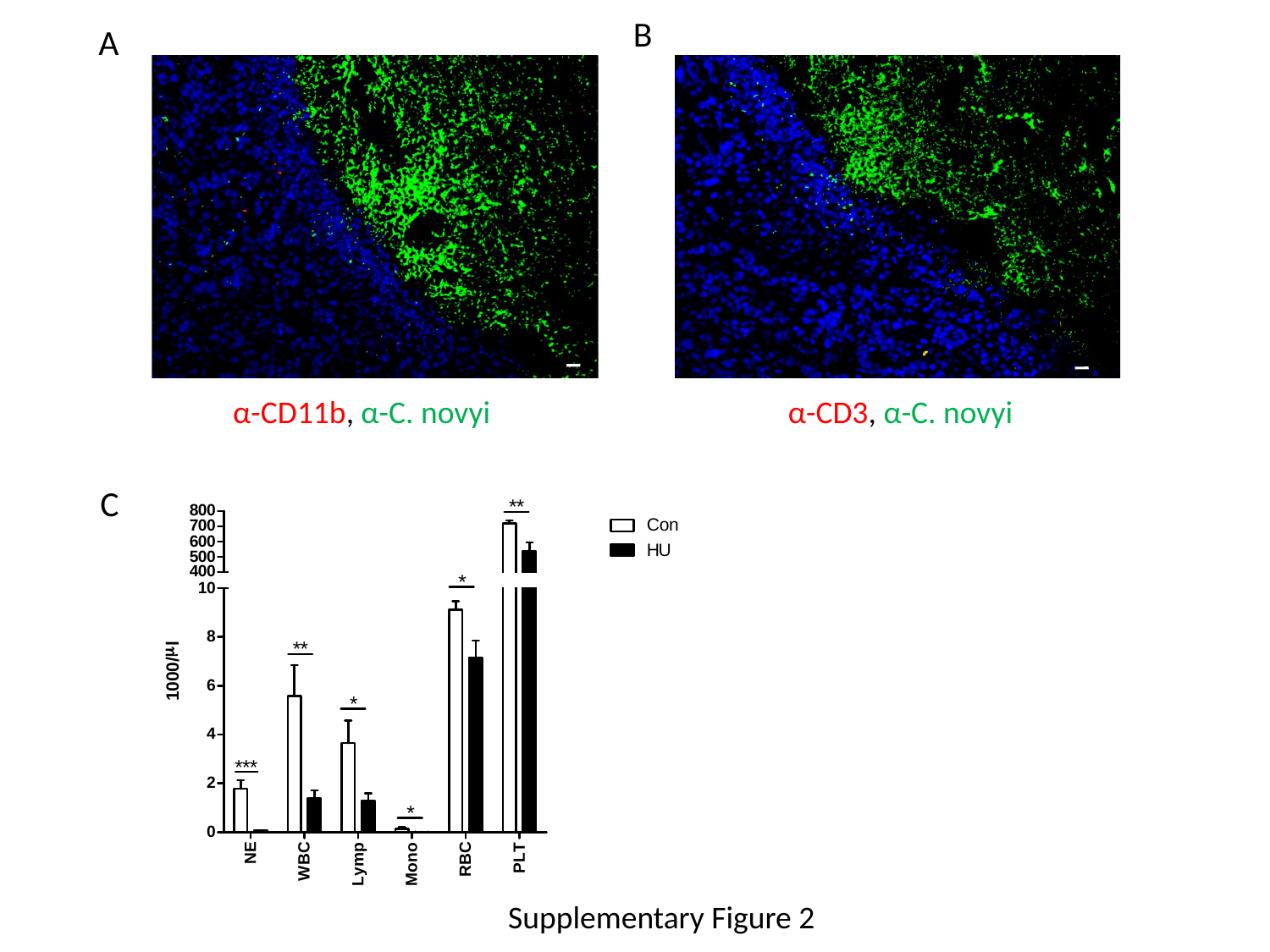

B
A
α-CD11b, α-C. novyi
α-CD3, α-C. novyi
C
Supplementary Figure 2

## Slide 3
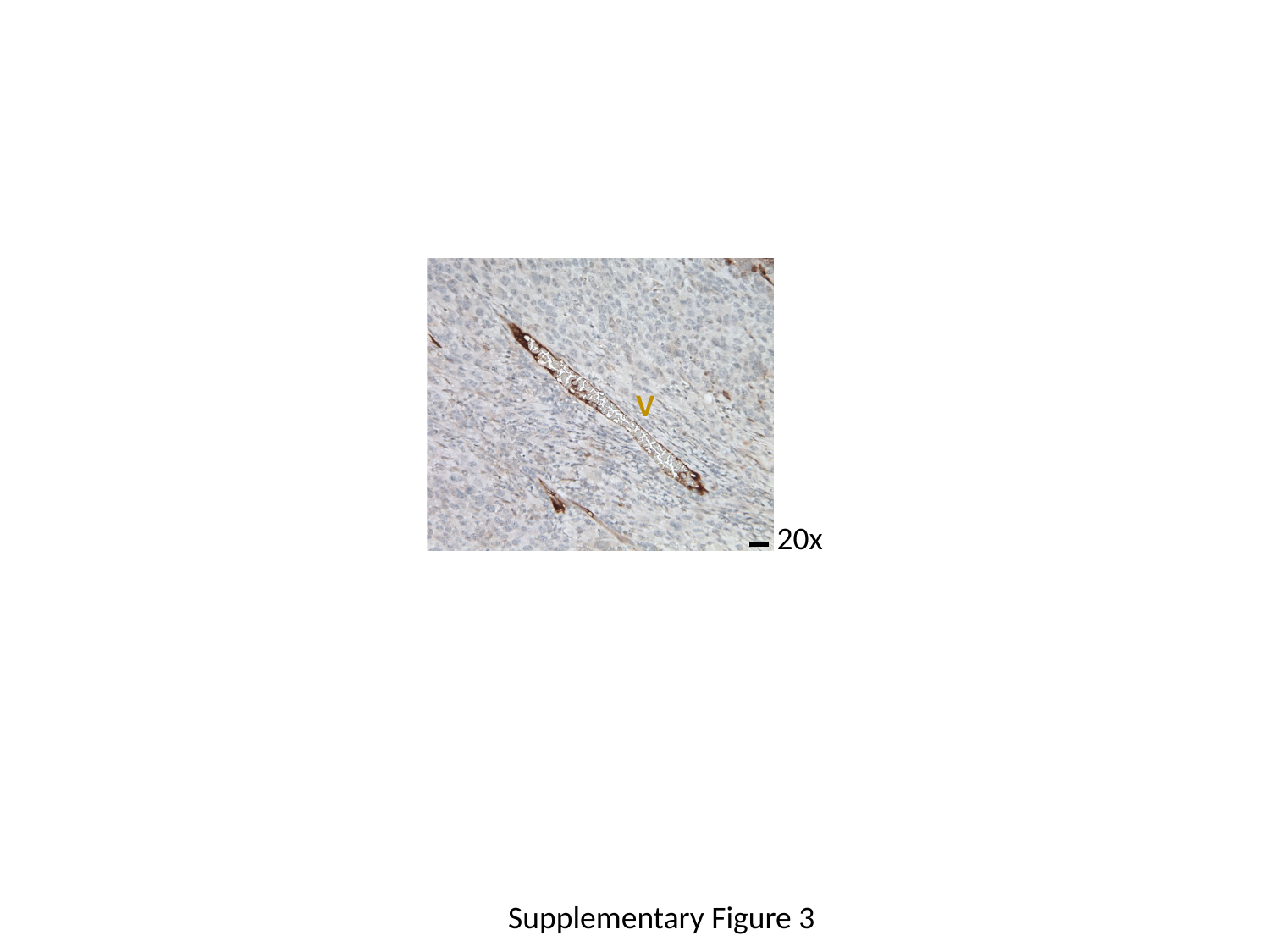

V
20x
Supplementary Figure 3

## Slide 4
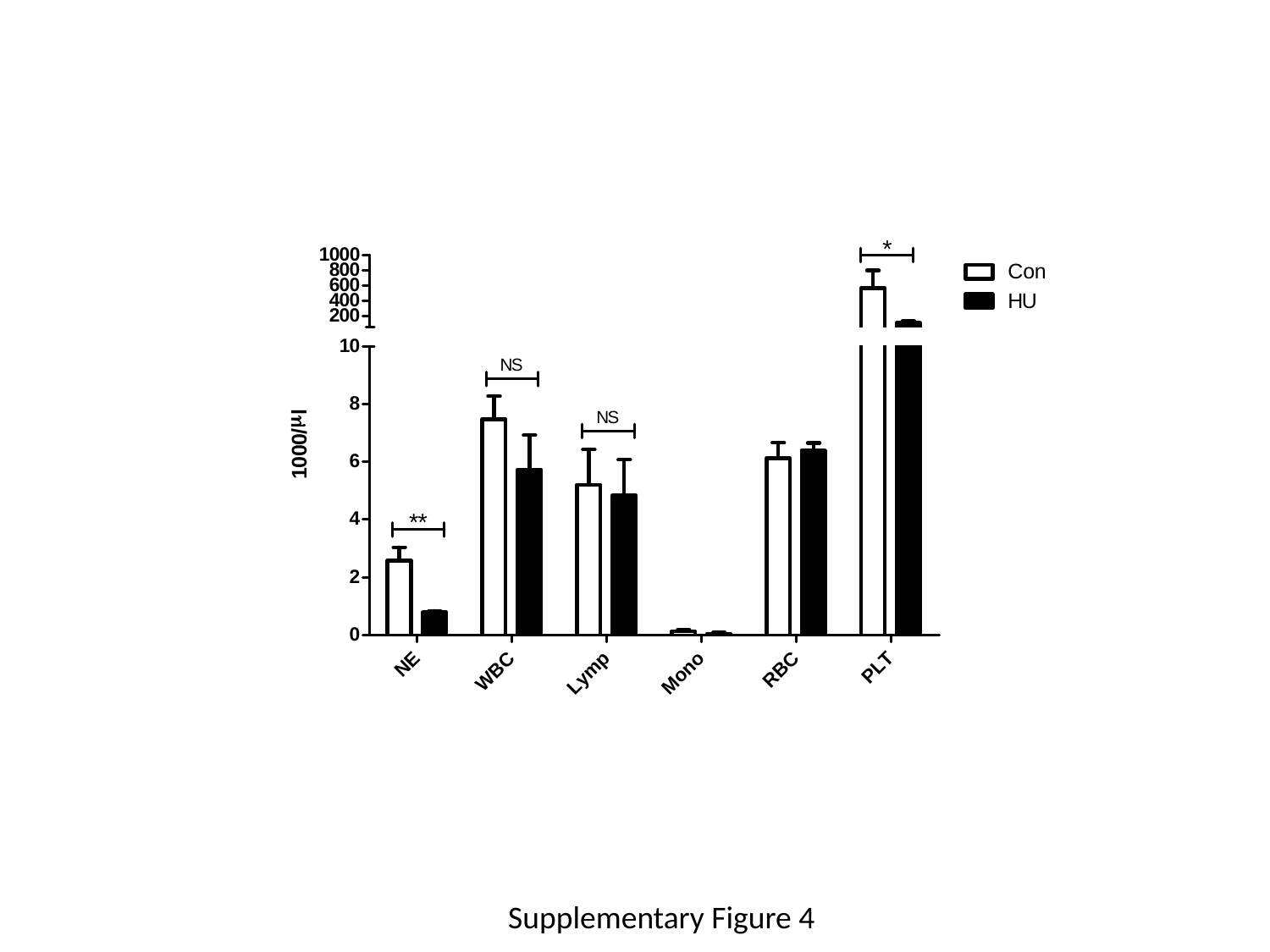

Supplementary Figure 4

## Slide 5
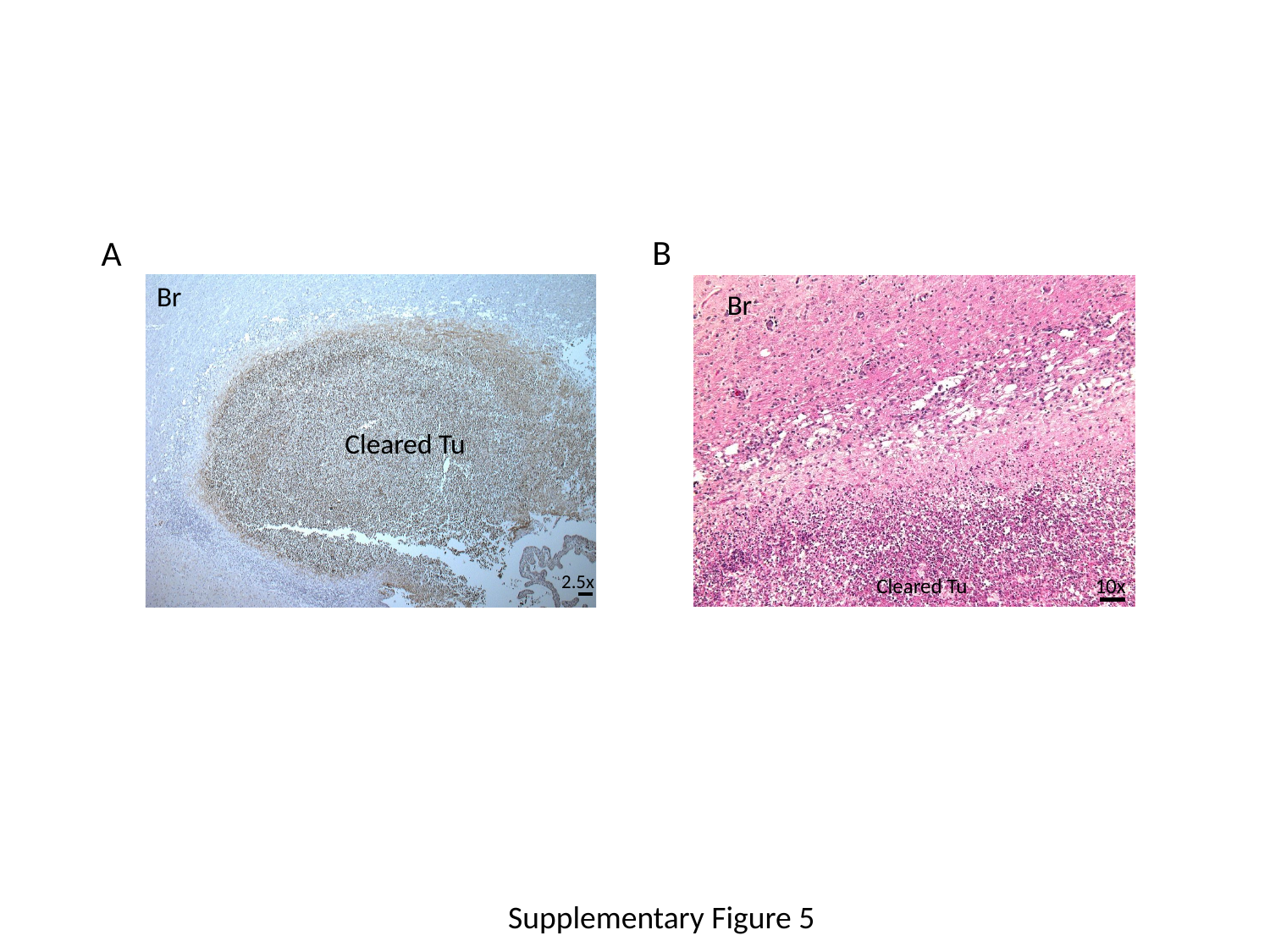

B
A
Br
Br
Cleared Tu
10x
Cleared Tu
2.5x
Supplementary Figure 5

## Slide 6
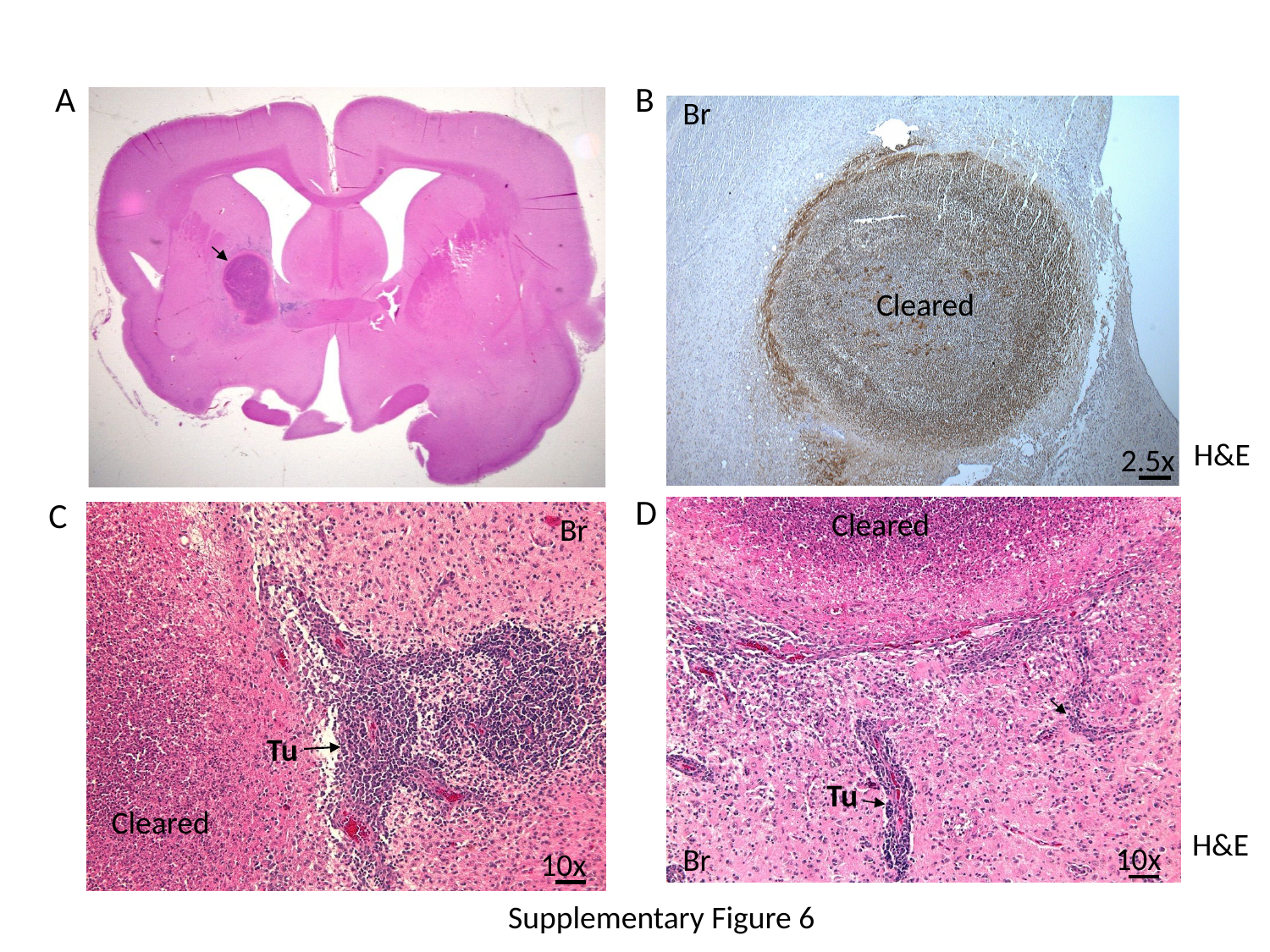

A
B
Br
Cleared
H&E
2.5x
D
C
Cleared
Br
Tu
Tu
Cleared
H&E
10x
Br
10x
Supplementary Figure 6
