# Supplementary material for: Neutrophil depletion enhanced the Clostridium novyi-NT therapy in mouse and rabbit tumor models
Source: Neurooncol Adv. 2021 Dec 21;4(1):vdab184. doi: 10.1093/noajnl/vdab184 (PMC8807082; doi:10.1093/noajnl/vdab184)
Supplement: vdab184_suppl_Supplementary_Figure_Legends [file vdab184_suppl_supplementary_figure_legends.docx]

**Supplementary Figure Captions**

**Supplementary Figure 1**

**A**. Flank GL261 tumor was injected with *C.novyi*-NT spores and harvested 24 hrs after the spore injection and Gram staining with safranin counter-staining demonstrated germinating bacteria (blue) and marked accumulation of polymorphonuclear granulocytes between the germinating bacterial and remaining tumor rim. Tu: tumor. NE: neutrophil. Scale bar: 50µm.

**B**. Those polymorphonuclear granulocytes were depleted by treating the mice with 1A8 antibody intraperitoneally (IP) 24 hrs before the spore injection, shown by the Gram staining where the bacteria (blue) penetrated the tumor rim freely. Scale bar: 20µm.

**Supplementary Figure 2**

**A&B**. Flank GL261 tumor was injected with *C.novyi*-NT spores and harvested 24 hrs after the spore injection. *C.novyi*-NT was stained green and staining with anti-CD11b (macrophages and monocytes) and anti-CD3 (T cells) antibodies (red) revealed minimal positive cells among the immune cell accumulation. Scale bar: 20µm.

**C**. Blood counts of mice treated with HU (n=3). HU treatment drastically reduced the neutrophil counts, while other blood cells were also affected in various degrees by HU.

**Supplementary Figure 3**

Flank GL261 tumor was labelled with pimonidazole (PMN) via IP injection 90 min before harvesting and immunohistochemistry staining of PMN showed positive staining (brown) of tumor blood vessel structures. This is a higher magnification (20x) of Fig. 2A. Scale bar: 20µm.

**Supplementary Figure 4**

Rabbits were treated with HU for 5 days and blood cell counts were compared with the control rabbits (n=3). HU treatment induced a significant reduction of neutrophil counts, and in lesser degree also the platelets, but has not affected other blood cell types significantly.

**Supplementary Figure 5**

**A.** A macroscopic overview of former brain tumor area of rabbit treated with HU and *C.novyi*-NT spores and harvested 8 days after the treatment in a healthy condition. Section was stained with anti-MPO antibody (brown) and showed the lesion was filled with neutrophils. Scale bar: 100µm.

**B.** An H&E staining of this brain section showed tumor was cleared 8 days following *C.novyi*-NT treatment. Scale bar: 50µm.

**Supplementary Figure 6**

A rabbit treated by *C.novyi*-NT alone survived the initial germination and was sacrificed at healthy condition 8 days after the spore injection.

**A**. A macroscopic view of the coronal H&E section of the brain revealed a restricted lesion (black arrow).

**B**. The brain section was stained with anti-MPO IHC, a neutrophil enzyme. The lesion was filled predominantly with neutrophils without germinating bacteria. Cleared: area where tumor cells were completely eradicated. Scale bar: 200µm.

**C&D**. Growing tumor cells were observed in the former tumor-brain transition areas, indicating a partial clearance of the tumor. Scale bar: 50µm.
